# Evaluation of the psychometric properties of a modified version of the Social Phobia Screening Questionnaire for use in adolescents

**DOI:** 10.1186/1753-2000-3-36

**Published:** 2009-11-11

**Authors:** Malin Gren-Landell, Andreas Björklind, Maria Tillfors, Tomas Furmark, Carl Göran Svedin, Gerhard Andersson

**Affiliations:** 1 Linköping University, Department of Clinical and Experimental Medicine, Linköping, Sweden; 2 Linköping University, Department of Behavioral Sciences and Learning, The Swedish Institute for Disability Research, Linköping, Sweden; 3 Örebro University, School of Law, Psychology and Social work, Örebro, Sweden; 4 Uppsala University, Department of Psychology, Uppsala, Sweden; 5 Karolinska Institutet, Department of Clinical Neuroscience, Stockholm, Sweden

## Abstract

**Background:**

Social phobia (social anxiety disorder - SAD) is a rather common but often undetected and undertreated psychiatric condition in youths. Screening of SAD in young individuals in community samples is thus important in preventing negative outcomes. The present study is the first report on the psychometric properties of the Social Phobia Screening Questionnaire for Children and adolescents (SPSQ-C).

**Methods:**

The SPSQ-C was administered to a community sample of high-school students. Test-retest reliability over three weeks was evaluated (n = 127) and internal consistency was calculated for items measuring level of fear in eight social situations. To measure concurrent validity, subjects who reported SAD on at least one occasion and randomly selected non-cases were blindly interviewed with the Structured Clinical Interview for DSM-IV Axis-I disorders (SCID-I), as gold standard (n = 51).

**Results:**

A moderate test-retest reliability, *r *= .60 (*P *< .01), and a satisfactory alpha coefficient of .78 was found. Values of sensitivity and specificity were 71% and 86% respectively, and area under the curve (AUC) was .79. Positive likelihood ratio (LR+) showed that a positive screening result was five times more likely to be correct than to reflect a non-case. Negative likelihood ratio (LR -) was .34. In addition, positive predictive value was 45% and negative predictive value was 95%. The prevalence of self-reported SAD was found to be 7.2% at the first assessment.

**Conclusion:**

The SPSQ-C is a short and psychometrically sound questionnaire for screening of SAD in adolescents, with the advantage of being based on the DSM-IV criteria.

## Background

Social anxiety disorder, also called social phobia, is a rather common anxiety disorder in adolescents, though prevalence rates are varying due to methodological and cultural reasons as well as due to what age groups are studied [1-4]. For many young sufferers, it is a disabling condition associated with a significantly increased risk for negative outcomes like dropping out from school [5], depression and suicide [6,7], alcohol use disorder [8] and cannabis dependence [9].

Even though effective psychosocial and pharmacological treatments for childhood SAD exist [10-13] help-seeking is low [4,14,15]. Children are usually referred to mental health service via parents but SAD is rarely recognized by parents and teachers [16] and mental health referral and treatment utilization is lower in anxiety disorders than in externalizing disorders in children and adolescents [17,18]. If help is sought, identification of symptoms needs to take place before treatment can be offered. While SAD is common in primary care populations, it is often not detected by primary care providers [19]. The use of a reliable and valid, brief screening instrument in primary care paediatric settings can facilitate the detection of SAD in adolescents [20]. According to the Practice parameters for anxiety disorders in children and adolescents [21], routine screening for anxiety symptoms is recommended during the initial mental health assessment due to the high prevalence of anxiety disorders. Also, given the high rates of comorbidity among anxiety disorders, there is a need to correctly identify the primary diagnosis, and rule out phenomenologically similar conditions that may be of importance for treatment selection [22]. The Practice parameters recommend that screening questions are based on DSM-IV criteria [23] and use developmentally appropriate language.

There are a few psychometrically evaluated self-report instruments for use in the assessment of SAD in children and adolescents. The most widely used and well established instruments are the Social Anxiety Scale for Children - Revised (SASC-R) [24], the Social Anxiety Scale for Adolescents (SAS-A) [25] and the Social Phobia and Anxiety Inventory for Children (SPAI-C) [26]. The SPAI-C has also been evaluated in a shorter 16-item version [27]. In addition, the Screen for Child Anxiety Related Emotional Disorders (SCARED) [28] can be used for the assessment of social anxiety disorder in children. The Social Phobia Inventory (SPIN) [29-31] has a more categorical format and has primarily been used with adolescents. The SPIN and the SPAI-C have been developed from instruments that have been used in adults, as well as an established Swedish screening instrument for use in adults, the Social Phobia Screening Questionnaire (SPSQ) [32]. The SPSQ has shown excellent psychometric properties, showing a sensitivity of 100% and specificity of 95%, and has been used in several epidemiological and treatment studies on adults [32-37]. The SPAI-C mentioned above, has been translated and evaluated in a Norwegian sample [38], but to date there is no validated instrument for screening of social anxiety in Swedish children and adolescents. In backdrop of the need of a brief, DSM-based screening questionnaire for use with Swedish children and adolescents, a modified version for children and adolescents, the Social Phobia Screening Questionnaire for children and adolescents (SPSQ-C), has been developed and used in epidemiological and descriptive studies of children ranging in age from twelve to eighteen years [1,39].

While the SPSQ-C is a time-efficient and potentially useful instrument based on DSM-IV criteria, it has yet to be psychometrically evaluated. Thus, the objective of the present study was to report preliminary results of the psychometric properties of the SPSQ-C in a community sample of high-school students. Reliability was investigated by test-retest analysis over a three-week period and by calculating internal consistency for the first eight items of the SPSQ-C, covering level of fear in different social situations. Concurrent validity, i.e. sensitivity and specificity of the questionnaire, was evaluated using the Structured Clinical Interview for the DSM-IV Axis I-disorders (SCID-I) [40] as gold standard.

## Methods

### Procedure

Data were collected on three occasions. On the first two, the SPSQ-C was used for the purpose of evaluating reliability and on the third occasion a clinical interview was used for establishing concurrent validity.

Two weeks before the investigation took place written information about the study were mailed to students and their parents. The students were also informed about voluntary participation at all three assessments. Data-collection for the reliability evaluation was done at the classes' weekly class-council. Students signed written consent, completed the screening questionnaire and answered additional questions regarding socio-demographics. The same procedure, with the same classes of students, was used three weeks later. As a compensation for their participation, the students had a chance of winning a ticket to a movie in a lottery that was conducted in each class after all students had completed their questionnaires at the first and second assessment.

A case-control design was adopted for the evaluation of validity. The procedure of a case-control study starts with the selection of known cases and then an appropriate number of controls are selected [41]. One week after the last assessment, adolescents meeting the criteria for social phobia according to the SPSQ-C, were selected if they had reported SAD on at least one occasion except if reporting SAD at the first assessment but not the second. Non-cases were randomly selected for the control group.

The clinical interview was conducted by telephone by two interviewers who were blind to the participants' diagnostic status on the SPSQ-C. A telephone format was chosen due to that many of the students were living in geographically distant areas, leading to transportation difficulties. Telephone administration of structured clinical interviews has been found to yield reliable, valid and time-effective data in the assessment of anxiety disorders in children [42]. Subjects were compensated for their participation in the interview, by movie-tickets. The study was approved by the local ethics committee.

### Subjects

#### Total sample

Subjects were recruited from a compulsory high school, in a small municipality (12 000 inhabitants) in the south middle of Sweden. The students were following the high school Social Science Programme or the Child Recreation Programme. These two programmes were chosen in order to have students from a theoretically oriented and a practically oriented programme.

In order to obtain a sample of ten subjects reporting SAD, as a minimum for the statistical analyses, a convenience sample of 180 subjects from eight classes (year 1-3) was selected. The size was due to an estimated prevalence rate of 4-14% of SAD in adolescents [1,2,14,43] and an expected absent rate of 10-15% on one school day [44]. The response rate at the first assessment was 85% and 79% at the second, resulting in a total of 169 subjects participating at any of the assessments. The subjects in the total sample were in the 1^st ^(n = 62), 2^nd ^(n = 67) and 3^rd ^(n = 40) year of studies. Mean age was 16.8 years (range 15-18 years). See Table 1 for further demographics of the total sample.

**Table 1:** Socio-demographics of the total sample (N = 169).

Age*	n *(%)*
15	2 (1.2)
16	63 (37.3)
17	61 (36.1)
18	42 (24.9)
**Gender**	
Male	100 (59.2)
Female	69 (40.8)
**Birth of origin**	
Swedish	156 (92.3)
Foreign	13 (7.7)
**Parents' birth of origin**	
Swedish, both	150 (88.9)
Foreign, one parent	9 (5.3)
Foreign, both	10 (5.9)
Living arrangement*	
With parents	136 (80.5)
With non-family**	31 (18.3)
Alone	**1 (0.6)**

*data missing in one case

** living with friend, partner or at boarding-school

#### Reliability sample

At the first assessment (n = 153) 89 boys, (58%) and 64 girls (42%) participated and at the second assessment (n = 143), 88 boys (61%) and 55 girls (39%). A total of 127 subjects participated at both measurements with the SPSQ-C and data from these subjects were used for the analysis of test-retest reliability.

#### Validity sample

In the present study a sample size of fifty subjects was chosen in order to have enough power for the evaluation of validity. Thirteen subjects reported SAD at both assessments or at one if only participating at one occasion and were eligible for the validity study (6/13 subjects had participated at both assessments and seven at one assessment). In order to get a sample of fifty subjects, thirty-eight non-cases were blindly and randomly selected by a person who was not involved in the project. A total of fifty-one subjects (26 males and 25 females) were interviewed. Seven subjects declined to participate and were substituted by the next numbered subject on the list for randomized selection. Non-responders consisted of one subject who reported SAD on the SPSQ-C and seven subjects who had not reported SAD. The non-responders were all male from the second year of their social science studies.

### Instruments

#### The Social Phobia Screening Questionnaire for Children and adolescents (SPSQ-C)

The SPSQ-C is a modified version of the Social Phobia Screening Questionnaire (SPSQ) for adults [32]. The SPSQ has shown satisfactory psychometric properties; an alpha coefficient of .90 concerning the section with fear ratings and high values of sensitivity and specificity [32].

The diagnostic section of the SPSQ-C is based on 8 potentially phobic situation: "speaking in front of the class", "raising your hand during a lesson", "being together with others during breaks", "initiating a conversation with someone one does not know very well", "looking someone in the eyes during a conversation", "making a phone-call to someone one does not know very well", "going to a party", and "eating together with others during the lunch-break". The respondents rate their perceived social fear in these potentially phobic situations on a three-point scale corresponding to no fear, some fear, and marked fear. Five diagnostic questions follow, assessing whether the individual meets the DSM-IV social phobia criteria A, B and D for one or more of the phobic situations. Since the instrument is developed for adolescents up to the age of 18, the C-criteria, realizing that the fear is excessive or unreasonable, does not have to be fulfilled. The E-criterion is assessed with three yes/no questions, i.e. the student is asked whether the social fear is of such nature that it severely interfere with or severely interfered with his/her activities in school, during leisure-time or when being with peers. The last question covers the F-criterion of 6-month duration (yes/no question). Criteria G (the fear is not due to direct physiological effects of a substance or medical condition, and not better accounted for by another mental disorder) and H (if a general medical condition or another mental disorder is present, the social fear is unrelated to it) are not assessed.

In order to establish a diagnosis of SAD on the SPSQ-C, i.e. a probable case of SAD, the student had to rate at least one potentially phobic situation as "marked fear" on the social fear scale. This particular situation had to be consistently endorsed in the diagnostic questions covering social phobia criteria A, B and D. The E-criterion had to be met, i.e. the report of impairment in at least one of the three life domains assessed. Lastly, the F-criterion, concerning persistence of symptoms for more than six months, also had to be fulfilled.

The SPSQ-C can be used dimensionally to determine subtypes of SAD and to measure severity of social anxiety. In the present paper, only data on a categorical level is presented. Different cut-off levels have beentested in the development phase of the SPSQ-C [1] and this was also done when the adult version of the SPSQ was developed [32]. The cut-off used is the closest to adhere to the DSM-IV definition of social phobia.

A paper and pencil format of the SPSQ-C was used. The instrument took about 5-10 minutes to fill out.

#### The Structured Clinical Interview for DSM-IV Axis 1 Disorder (SCID-1)

To evaluate concurrent validity, the SPSQ-C was compared with the SCID-I [40] used as gold standard. For the purpose of this study, only the section covering SAD in the research version of the SCID-I was used. The social phobia section of the SCID has previously been used in a telephone format with students from the age of 17 [45]. The interviews were made by a student in the last year of his master graduation of psychology studies with basic training in the diagnostic procedures and by a mental health professional with long experience in using rating scales and diagnostic interviews in clinical and research contexts. The mental health professional conducted 35 of the 51 interviews. The respondents were interviewed by telephone and the interview took 5-20 minutes to conduct. The interviewers were blind to the subjects' response on the SPSQ-C.

### Statistical analyses

Chi-square or Fisher's exact tests were used for evaluating group differences with respect to categorical variables. Test-retest reliability was assessed using Spearman's correlation coefficient. The internal consistency of the scale was assessed using the Cronbach's coefficient alpha for the first eight items of the SPSQ-C (data from the first assessment). Specificity (1-α) and sensitivity (1-β), positive and negative likelihood ratios were calculated as well as positive predictive value (PPV) and negative predictive value (NPV). All analyses were performed in SPSS version 15.0 (SPSS, Inc., Chicago, IL, USA).

## Results

### Descriptives

At the first measurement (n = 153) eleven subjects (7.2%) met the criteria for SAD according to the SPSQ-C (4.5% of the males and 10.9% of the females) and 7.7% (4.5% of the males and 12.7% of the females) at the second measurement (n = 143). There was no significant difference between the genders in reporting SAD on the SPSC-Q neither at the first measurement (χ^2 ^= 2.32, df = 1, = ns) or the second (χ^2 ^= 3.19, df = 1, = ns). No significant differences were found between cases and non-cases on any of the demographic variables.

### Measures of reliability

The alpha coefficient for the first items on eight phobic situations in the SPSQ-C was .77. Reliability test-retest analysis yielded a correlation of *r *= .60 (*P *< .01) between the two assessments. In addition, we also calculated an intraclass correlation (ICC) and a significant correlation coefficient of .75, was found.

### Measures of validity

**Figure 1:**
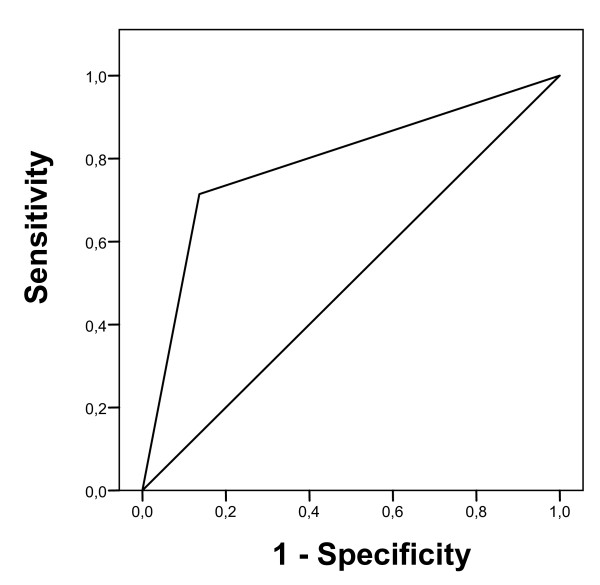
**Receiver operation characteristics (ROC) curve for the SPSQ-C**.

The overall test accuracy, i.e. the percentage of correct diagnoses in the validity sample, was 84%. The area under the curve (AUC) was .79 which was significant in comparison to a random ROC line (*P *< .015), see Figure 1. ROC-analysis showed sensitivity to be 71% and specificity 86%. This means that 71% of the respondents who were screened positive on the SPSQ-C were diagnosed with SAD on the SCID-I (5/7), and that 86% (38/44) scored negative on the SPSQ-C and were not diagnosed with SAD on the SCID-I. Accordingly, the positive likelihood ratio (LR+) was 5.07. This means that a self-reported case of SAD is about 5 times more likely to be a true case than a non-case. The negative likelihood ratio (LR-) was .34. This means that a negative screen on the SPSQ-C is marginally likely to identify a true non-case.

Predictive values represent the probability of an outcome after the results are known. In the present study, positive predictive value (PPV), the percentage of positive screens that are accurate, was 45% (5/11). Negative predictive value (NPV), i.e. the percentage of respondents screening with a negative test result who were not diagnosed with SAD, was 95% (38/40).

## Discussion

The aim of the present study was to evaluate the psychometric properties of a screening questionnaire for SAD in a community sample of Swedish adolescents. Firstly, satisfactory reliability was found. Concerning internal consistency, an alpha coefficient should be at least .60 for a self-report instrument to be reliable [46]. In the present study an alpha coefficient of .78 was found, showing that the eight items on the SPSQ-C are highly internally consistent and that the items appear to measure a common structure. In measuring test-retest reliability, we found a positive correlation of temporal stability over a three week period of *r *= .60. In measuring reliability, values of .50 to .70 are considered moderate [47]. Studies of other self-report measurements of SAD or social anxiety show long-term and short-term test-retest correlations ranging from .47 to .86 [26,29,48]. The test-retest reliability and intra-class correlation of SPSQ-C is thus by and large comparable to those of well-established measures in use for the assessment of SAD in children and adolescents.

Secondly, concurrent validity was assessed, yielding a specificity of 86% and a sensitivity of 71%. These values are comparable to other instruments screening for symptoms of social anxiety [20]. Sensitivity values of at least 70% are considered essential [49]. The greater value of specificity, the more cost-efficient is the instrument and a specificity value above 80% is considered useful [49]. The AUC was .79. Values of .70-.80 are considered fair and >.80 as good [20,50]. In determining the optimal cut-off point, it has been suggested that the costs of false positives and false negatives should be considered [51]. In the present study we did not calculate cut-off scores based on cost-efficiency.

In addition to evaluating sensitivity and specificity, it is of clinical interest to describe predictive values. The negative predictive value was 95%, i.e. the probability that SPSQ-C correctly identifies individuals with no SAD. We found a positive predictive value of 45%. The predictive values are influenced by prevalence rates and low prevalence rates produce higher NPV and lower PPV. In the present study a prevalence rate of 7.2% was found at the first assessment and 7.7% at the second assessment.

There are some limitations to be mentioned in relation to the results from the present study. First, only concurrent validity was assessed. For clinical purpose, it would be of value to differentiate SAD from other clinical conditions but in the present study discriminant validity of the SPSQ-C was not investigated. Symptoms of anxiety are part of normal development and screening instruments need to have the ability to discriminate those with disabling symptoms from those within normal levels of worry and anxiety [47]. Thus, the SPSQ-C should be evaluated in comparisons with other instruments and behavioral assessment. Detection of social anxiety needs to take place early in order to prevent the development of further mental illness. Thus, it is of interest to evaluate the SPSQ-C in a community sample in the first place. It is also of interest to evaluate the SPSQ-C in clinical groups and to study the instrument's ability to measure severity and treatment efficacy [22,52]. Further studies of the SPSQ-C should include the evaluation of convergent validity by comparing the SPSQ-C to other self-report measures.

Second, the subjects in the present study were high-school students. Onset of SAD is usually in early- to mid-adolescence but has been diagnosed in children as young as 7-8 years-old [53]. Assessment methods should be developmentally sensitive [21,52,54]. There are difficulties in developing questionnaires that are suitable for different ages [55] and little work has been done on early identification and assessment of social anxiety in children [54]. In this first report only adolescents were included but psychometric evaluation in younger age groups is needed.

A third limitation is the small sample size. The power of the statistical analyses would have increased by a larger number of subjects.

Lastly, recruitment of participants for the evaluation of validity was made from two different assessments. This was done in order to make ecological use of data but it also results in variability between subjects regarding the time-span between the measurements with SPSQ-C and the SCID-I.

As a final comment, better detection of social anxiety disorder is not a goal in itself, i.e. screening should be done only when further assessment, treatment and follow-up also is offered [56]. Unfortunately, there are frequently barriers to treatment utilization [57,58] and little is known on how to increase mental health utilization among socially phobic individuals [59]. Finding methods that could make treatment available for socially anxious children and adolescents remains a challenge.

## Conclusion

Screening of SAD in adolescents is critical for prevention and treatment. Compared to other self-reports questionnaires, the SPSQ-C has the advantage of being a short and cost-efficient screening instrument, based on the DSM-IV criteria of social anxiety disorder including measures of impairment and duration of SAD but also measures on a dimensional level. The results lend support to that it is a reliable and valid screening device for non-clinical older adolescents.

## Competing interests

The authors declare that they have no competing interests.

## Authors' contributions

MGLl planned the design of the study, took part in collecting data, analysed data and was primarily responsible for writing the manuscript. AB planned the design of the study, collected data and conducted the analyses, took part in reading the ms and approved to the final version of the ms. TF developed the SPSQ-C, took part in the preparation of the manuscript and made major contributions to the manuscript including language revision. MT developed the SPSQ-C, took part in the statistical analyses, discussion of the design and in the preparation of the ms. CGS supervised the design and execution of the study and made contributions to the ms.*GA *supervised the design and execution of the study and made contributions to the ms. All authors have read and approved the final ms.
